# Prenatal Exposure to Gestational Diabetes Mellitus is Associated with Mental Health Outcomes and Physical Activity has a Modifying Role

**DOI:** 10.21203/rs.3.rs-3290222/v1

**Published:** 2023-08-29

**Authors:** Jasmin M. Alves, Alexandro Smith, Ting Chow, Sonya Negriff, Sarah Carter, Anny H. Xiang, Kathleen A. Page

**Affiliations:** University of Southern California; University of Southern California; Kaiser Permanente Southern California; Kaiser Permanente Southern California; Kaiser Permanente Southern California; Kaiser Permanente Southern California; University of Southern California

**Keywords:** developmental programming, GDM exposure, anxiety, depression, physical activity, childhood

## Abstract

**Background:**

Studies suggest a link between prenatal gestational diabetes mellitus (GDM) exposure and poor mental health outcomes. We examined associations between prenatal GDM exposure and depressive and anxiety symptoms in children and assessed physical activity as a potential modifier of these associations.

**Method:**

Seventy children (Age_M(SD)_: 12(2.0), 56% GDM, 59% female) and their parents completed surveys: Center for Epidemiological Studies Depression Scale for Children (CES-DC), State-Trait Anxiety Inventory for Children (STAIC), Child Behavior Checklist (CBCL), and 3-day physical activity recall (3DPAR). Associations between mental health measures with GDM exposure and interactions between GDM exposure and child moderate-to-vigorous physical activity (MVPA) were assessed using regression.

**Results:**

GDM-exposed children had higher anxiety (p = 0.03) and internalizing symptoms (CBCL) (p = 0.04) than unexposed children. There was an interaction between GDM exposure and child MVPA on anxiety (p = 0.02), internalizing (p = 0.04) and externalizing symptoms (p = 0.004). In the low MVPA group, GDM exposed children had more depressive (p = 0.03), anxiety (p = 0.003), and internalizing symptoms (p = 0.03) than unexposed children. In the high MVPA group, there were no group differences except with externalizing symptoms (p = 0.04).

**Conclusion:**

Prenatal GDM is associated with higher anxiety and internalizing symptoms in children. Child MVPA modified the relationship between GDM exposure and mental health outcomes suggesting that physical activity during childhood could mitigate the negative mental health outcomes associated with prenatal GDM exposure.

## Background

The prevalence of depression and anxiety among youth in the United States continues to rise (Ghandour et al., 2019; Kalb et al., 2019). Growing evidence suggests that an important contributor to childhood mental health outcomes is prenatal exposure to gestational diabetes mellitus (GDM) (Nahum Sacks et al., 2016; Nogueira Avelar e Silva et al., 2021). Rates of GDM also continue to rise with almost 7% of pregnancies impacted (Shah et al., 2021). Recent epidemiological studies have found that offspring exposed to maternal diabetes *in utero* have more than a 2-fold risk of being diagnosed with an anxiety disorder (Kong et al., 2018; Nogueira Avelar e Silva et al., 2021). However, findings have been mixed on whether GDM exposure is associated with increased depressive symptoms, with one study showing no associations with depressive symptoms (Yamasaki et al., 2019) and another showing that GDM exposure was associated with increased inattention and depressive symptoms during childhood (Nomura et al., 2012). Childhood is a critical time-period when anxiety and depression first begin to manifest (Beesdo et al., 2009; de Girolamo et al., 2012). Therefore, determining if prenatal exposure to GDM is associated with increased anxiety or depressive symptoms during childhood could be important for identifying potential preventive strategies to mitigate the development of anxiety and depressive disorders.

Several lines of research suggest that engaging in physical activity is associated with lower anxiety and depressive symptoms (Bélair et al., 2018; McMahon et al., 2017; Parfitt et al., 2009; Sund et al., 2011). Randomized clinical trials during childhood have shown that aerobic exercise is effective in reducing anxiety and depressive symptoms (Baccouche et al., 2013; Crews et al., 2004; Fidelix et al., 2019; Melnyk et al., 2009). Further, longitudinal studies provide additional support that engaging in physical activity during childhood protects against poor mental health outcomes in adolescence (Hamer et al., 2020; Kandola et al., 2020; Sund et al., 2011). Similarly, prior epidemiological studies have shown that engaging in physical activity modifies the relationship between perceived stress and depressive symptoms in children, such that children with high perceived stress who were more physically active reported less depressive symptoms than less physically active children (Sibold et al., 2020; Sund et al., 2011). Therefore, we reasoned that physical activity could serve as a potential modifier of the relationship between prenatal exposure to GDM and offspring mental health. While existing studies have not examined physical activity (Grudzinski et al., 2019; Kong et al., 2018; Mina et al., 2017) as a modifier of *in utero* exposures and child mental health, our recent study showed that vigorous physical activity levels during the height of the COVID-19 pandemic partially mediated the association between prenatal GDM exposure and child state anxiety levels (Alves et al., 2021).

Given the limited research on the impact of GDM exposure on childhood mental health, we investigated the association between GDM exposure and depressive and anxiety symptoms (parent and child report). We additionally assessed child physical activity as a potential modifier of these associations.

## Methods

### Study Overview

Seventy children from the larger BrainChild study completed this ancillary study assessing mental health outcomes in relation to GDM exposure (Mean ± SD age 12.0 ± 2.0, 59% female, 56% GDM-exposed). BrainChild is a cohort of typically developing children recruited from Kaiser Permanente Southern California (KPSC) (Luo et al., 2021; Page et al., 2019). To be eligible to participate, children must have met the following criteria: 1) KPSC’s electronic medical records (EMR) documenting diagnosis of GDM or normal glucose tolerance during pregnancy; 2) children were the offspring of singleton uncomplicated full-term births; 3) children had no diagnosis of neurological, psychiatric, or significant medical disorders. The institutional review board at both KPSC (# 10282) and University of Southern California (USC) (# HS-14-00034) approved this study and parental written informed consent and child assent were acquired.

Participants completed the State-Trait Anxiety Inventory for Children (STAIC), and the Center for Epidemiological Studies Depression Scale for Children (CES-DC) questionnaires and parents completed the Child Behavior Checklist (CBCL) and the 3-day physical activity recall (3DPAR) during an in-person study visit at the Diabetes and Obesity Research Institute at USC.

### Exposure

Maternal GDM status was extracted from EMR. GDM diagnosis was based on laboratory glucose values confirming a plasma glucose level ≥ 11.10 mmol/L from a 50-g glucose challenge tests or at least 2 plasma glucose values meeting or exceeding the following values on the 100-g or 75-g oral glucose tolerance test: fasting, 5.27 mmol/L; 1 hour, 9.99 mmol/L; 2 hours, 8.60 mmol/L; and 3 hours, 7.77 mmol/L (“Standards of Medical Care in Diabetes – 2012,” 2012).

### Study Measures

The STAIC was used to assess child self-reported trait anxiety using the T-anxiety subscale (Spielberger & Edwards, 1973). The STAIC is a well-validated measure to assess child anxiety in a research setting and has been validated in 1551 children age 6–14 years old (Erford & Lutz, 2015; Spielberger & Edwards, 1973). The CES-DC was used to assess self-reported depressive symptoms and has previously been validated in children age 6 to 23 years (Faulstich et al., 1986; Fendrich et al., 1990). Parent reported internalizing symptoms and externalizing symptoms were assessed using the CBCL (Achenbach & Ruffle, 2000). Internalizing symptoms are based on the following sub scores: anxious/depressed, withdrawn/depressed, and somatic complaints. Externalizing symptoms are based on the following sub scores: rule-breaking behavior and aggressive behavior sub scores. The CBCL is validated for both clinical and research settings based on normative sample of 4220 children age 6 to 18 (Erford & Lutz, 2015).

The 3DPAR was used to assess time spent in moderate and vigorous physical activity (Pate et al., 2003). With the input of each child’s parent, a trained staff member asked participants to recall their activities from 7:00am to 12:00am in 30-minute increments for the previous three days. The participant was then asked to rate the intensity of each activity ranging from ‘light’, ‘moderate’, ‘hard’ to ‘very hard’. Each corresponding activity and intensity level was then converted to metabolic equivalences (METS) (Ainsworth et al., 2011). Activities with METs ≥ 3 were classified as moderate-to-vigorous physical activity (MVPA). Examples of moderate to vigorous physical activities include bike riding or swimming. The final output was the average minutes spent in MVPA per a day. The 3DPAR has previously been validated with accelerometers in pediatric populations (Dollman et al., 2015; Pate et al., 2003; Pavlidou et al., 2010; Powell et al., 2009).

### Statistical Analysis

T-tests for means, Wilcoxon two-sample test for medians, Fisher’s exact tests, and Chi-square tests for frequencies were used to test for differences in child age, sex, maternal pre-pregnancy BMI, maternal education, and income at birth between unexposed and GDM-exposed children and their mothers. Linear regression was used to test group differences for GDM exposed and unexposed children in anxiety, depressive, internalizing, and externalizing symptoms. Total scores for depressive symptoms, internalizing symptoms, externalizing symptoms and MVPA were not normally distributed and were therefore square root transformed to normalize their respective distributions. For interpretative purposes, anxiety, depressive, internalizing, and externalizing symptoms were standardized by their respective standard deviations. Child age, sex, socioeconomic status, and maternal pre-pregnancy BMI were included as covariates. Socioeconomic status was based on maternal education at birth extracted from each child’s birth certificate as a categorical variable (high school, some college and college and above) and income based on census tract of residence information. Income was based on 7 categories (<$30,000; $30,000-$49,999; $50,000-$69999; $70,000-$89999; >$90,000). Maternal pre-pregnancy BMI (kg/m^2^) was calculated from EMR based on clinic visits within 180 days of the last menstrual period.

Additional regression models were run to include MVPA levels as an interaction term with GDM exposure in association with mental health outcomes. MVPA was categorized as below or above the Centers for Disease Control daily recommended amount of MVPA for children, which is at least 60 minutes a day spent in MVPA (US Department of Health and Human Services, 2018). Models with significant interactions were then run stratified by MVPA levels to test for group differences between unexposed and GDM-exposed children in mental health outcomes, and the same covariates were included. A significance level of p < 0.05 was used. SAS (SAS Institute, Cary, North Carolina) was used for all the statistical analyses.

## Results

There were no differences in child and maternal characteristics between unexposed and GDM-exposed children (Table 1). In unadjusted analyses, mental health measures did not significantly differ between unexposed and GDM exposed children (Table 1).

In models adjusted for child age, sex, socioeconomic status, and maternal pre-pregnancy BMI, GDM-exposed children had significantly higher self-reported anxiety symptoms (unexposed Mean ± SE: 4.23 ± 0.20; GDM-exposed Mean ± SE: 4.79 ± 0.16, p = 0.03) and parent-reported internalizing symptoms (unexposed Mean ± SE: 1.45 ± 0.20; GDM-exposed Mean ± SE: 1.99 ± 0.16, p = 0.04) than the unexposed children (Table 2). Depressive symptoms were not significantly different between the two groups, although depressive symptoms tended to be higher among GDM-exposed (2.75 ± 0.16) than unexposed (2.29 ± 0.19) children (p = 0.07). There was no difference in externalizing symptoms between unexposed and GDM-exposed children (p = 0.69). In models further adjusted for maternal race and ethnicity, there were no longer significant group differences in anxiety (p = 0.09) and internalizing symptoms (p = 0.11) between GDM-exposed and unexposed children, although anxiety and internalizing symptoms remained higher in GDM-exposed children (Table 2).

**Figure 1:**
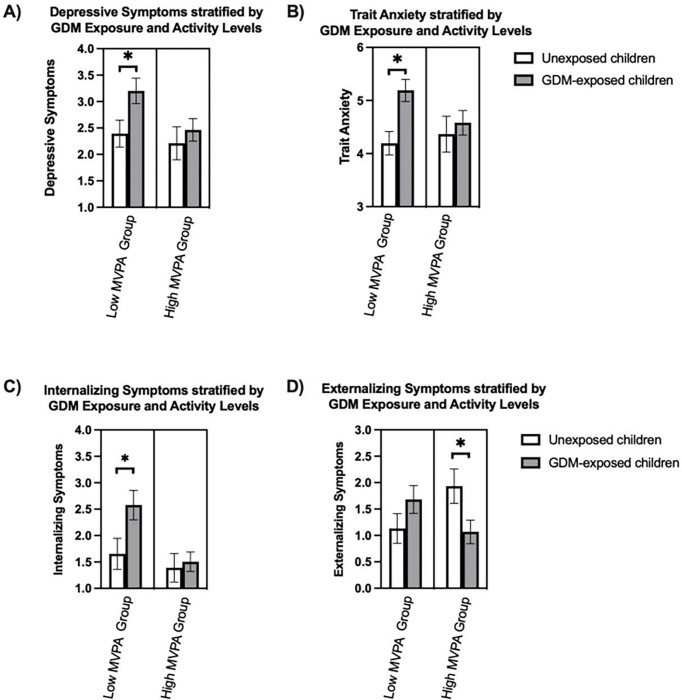
Association between Mental Health Outcomes and Physical Activity stratified by GDM Exposure Transformed LSmean Depressive Symptoms A), Anxiety Symptoms B), Internalizing symptoms C) and Externalizing Symptoms D) adjusted for child age, sex, socioeconomic status and maternal pre-pregnancy BMI. Moderate to vigorous physical activity (MVPA). Low MVPA group engaged in less than the recommended amount of MVPA for children, high MVPA group engaged in more than recommended amount of MVPA for children. Low MVPA unexposed (N=13), Low MVPA GDM-exposed (N=15), high MVPA unexposed (N=14), high MVPA GDM-exposed (N=19).

There was a significant interaction between MVPA and GDM exposure with trait anxiety (p = 0.008), internalizing symptoms (p = 0.02), externalizing symptoms (p = 0.01) and marginally with depressive symptoms (p = 0.06). These significant interactions remained after adjusting for child age, sex, socioeconomic status, maternal pre-pregnancy BMI and maternal race and ethnicity. In analyses stratified by MVPA levels, GDM-exposed children in the low MPVA group had more depressive symptoms (p = 0.04), trait anxiety (p = 0.004), and internalizing symptoms (p = 0.04) compared to unexposed children (Fig. 1, Table 3). In the high MVPA group, there were no differences in depressive symptoms, trait anxiety, or internalizing symptoms between unexposed and GDM-exposed children. In the high MVPA group, unexposed children had more externalizing symptoms (p = 0.04) compared to GDM-exposed children.

## Discussion

While prior studies have suggested that there is a link between intrauterine exposure to diabetes and increased risk for mental health disorders in offspring (Kong et al., 2018; Nogueira Avelar e Silva et al., 2021), this is the first study that has attempted to identify potential therapeutic targets to ameliorate negative mental health outcomes in GDM-exposed children. We showed that prenatal GDM exposure is associated with higher anxiety and depressive symptoms in children, and that child physical activity levels have a modifying role in these associations.

We previously found that during the beginning of the COVID-19 pandemic, GDM-exposed children exhibited higher state anxiety compared to unexposed children (Alves et al., 2021). Similarly, a recent epidemiological study also found that in 2,413,355 individuals, offspring exposed to GDM were 22% more likely to develop an anxiety disorder than those who were unexposed to GDM (Nogueira Avelar e Silva et al., 2021). The current study built upon these prior findings and showed associations between GDM exposure and parent-reported symptoms of child anxiety and depressive symptoms along with child-reported trait anxiety symptoms. In animal models, prenatal exposure to maternal diabetes invokes abnormal behavior and increased inflammation in the hippocampus, a brain region important for emotional regulation (Bannerman et al., 2004; Chandna et al., 2015; Piazza et al., 2019), suggesting that central nervous system inflammation may play a role in the association between prenatal exposure to maternal diabetes and poor mental health outcomes in offspring. Additional studies in rodent models have shown that prenatal exposure to maternal diabetes leads to altered insulin and insulin-like growth factor one (IGF-1) signaling (Fordjour et al., 2021; Jing et al., 2014), which may be linked to greater anxiety-like behaviors ^43^. It is worth noting, after adjusting for maternal race and ethnicity, we no longer observed significant associations between child mental health outcomes and GDM exposure, suggesting that maternal race and ethnicity may play a role in these associations. Future studies with a larger sample size are needed to examine the pathway by which maternal race and ethnicity are related to GDM exposure and child mental health outcomes.

In contrast, a number of studies have shown that increased physical activity is associated with favorable mental health outcomes (Gorham et al., 2019; Hamer et al., 2020; Kandola et al., 2020; Migueles et al., 2020; Sund et al., 2011). Our findings indicate that child physical activity has a modifying role in the association between prenatal exposure to GDM and mental health outcomes and this was independent of maternal race and ethnicity. In keeping with these findings, other studies have shown that physical activity is particularly beneficial for mental health outcomes among vulnerable youth (Baccouche et al., 2013; Sibold et al., 2020; Sund et al., 2011). For example, in a study of 13,583 high school students, physical activity modified the relationship between depressive symptoms and history of being bullied (J et al., 2015). Additional longitudinal studies in youth have observed that physical activity protects against future symptoms of depression and anxiety (Jerstad et al., 2010; Sund et al., 2011; Vella et al., 2017). Therefore, it is possible that engaging in physical activity protects against adverse mental health outcomes among children who may be at higher risk for developing mental health symptomatology, including GDM-exposed children.

While our study did not test specific mechanisms for the benefits of physical activity on offspring mental health, studies in rodents have shown that physical activity protects against anxiety and depressive symptoms through improvements in brain IGF-1 signaling (LLorens-Martín et al., 2010; Tai et al., 2020; Trejo et al., 2008), improved serotoninergic signaling (Matsunaga et al., 2021; Otsuka et al., 2016; Tai et al., 2020), hippocampal neurogenesis, and brain derived neurotropic factor (BDNF) production (Lapmanee et al., 2017; Taheri Chadorneshin et al., 2017; Uysal et al., 2015; van Praag et al., 1999). Therefore, there may be a variety of mechanisms by which physical activity could protect against poor mental health outcomes among GDM-exposed children, potentially by counteracting the negative effects of diabetes exposure on neural IGF signaling and/or inflammation. Future work is needed to examine the potential mechanisms by which physical activity may improve offspring mental health.

A strength of this study was inclusion of well validated assessments of both self-reported and parent-reported mental health measures. Additionally, GDM diagnoses were ascertained from electronic medical records. Our study also has limitations, including that maternal mental health measures were unavailable and could not be adjusted for in the analyses, which is similar to other studies investigating associations between child mental health outcomes and GDM exposure (Krzeczkowski et al., 2019; Nahum Sacks et al., 2016; Yamasaki et al., 2019). Future studies should consider the role of maternal mental health in these associations. Additional limitations include a relatively small sample size, physical activity that was assessed using questionnaires rather than objective measures such as accelerometers, and the correlational nature of the study which precludes causal interpretation. Future experimental studies are needed to test whether engaging in physical activity can ameliorate anxiety and/or depressive symptoms among GDM-exposed offspring, and to examine potential mechanisms underlying the benefits of physical activity on offspring mental health. Additionally, longitudinal studies that extend into adolescence, when the incidence of mental health problems increases, would provide important insight into the role of GDM exposure on mental health outcomes in offspring (de Girolamo et al., 2012). Longitudinal studies could also provide further evidence of a potential role of physical activity in protecting against poor mental health outcomes in children exposed to GDM *in utero*. As mental health concerns among youth continue to rise, identifying modifiable behaviors that ameliorate mental health outcomes amongst vulnerable youth is crucial (Ghandour et al., 2019; Kalb et al., 2019).

## Conclusions

We found that prenatal exposure to GDM was associated with increased anxiety and internalizing symptoms. Our findings lend further support to the role that prenatal exposure to GDM may have in offspring mental health outcomes. Further, we found that child physical activity modifies the relationship between prenatal exposure to GDM and offspring mental health outcomes. These results suggest that physical activity could be a potential therapeutic target to protect offspring exposed to GDM *in utero* against poor mental health outcomes.

## Figures and Tables

**Table 1:** Child and Maternal Characteristics

Variable	Unexposed (N = 31)	GDM-exposed (N = 39)	p-value**

**Child Characteristics**			

Mean Age (SD)	12.0 (1.9)	12.0 (2.1)	0.89

Sex: N (%)	Girls: 18 (58%)	Girls: 23 (59%)	0.94

Median Internalizing symptoms (IQR)	4 (2, 6)	5 (2, 11)	0.15

Median Externalizing symptoms (IQR)	2 (1, 6)	2 (0, 6)	0.59

Median Depressive symptoms (IQR)	10 (6, 16)	11 (7, 24)	0.23

Mean Anxiety symptoms (SD)	33.1 (7.2)	36.7 (8.0)	0.06

Median MVPA (IQR) (min)	50 (30, 90)	60 (20, 110)	0.71

**Maternal Characteristics**			

Mean Maternal pre-pregnancy BMI (kg/m^2^): (SD)	30.5 (7.0)	31.3 (7.7)	0.66

Family Income			0.41
<$30,000:	8 (26%)	5 (13%)	
$30,000–$49999:	$30,000–$49999: 9 (29%)	8 (21%)	
$50,000–$69,999:	$50,000–$69,999: 10 (32%)	15 (38%)	
$70,000–$89,999:	$70,000–$89,999: 3 (10%)	7 (18%)	
≥ $90,000:	≥ $90,000: 1 (3%)	4 (10%)	

Maternal Education	3 (10%)	10 (26%)	0.18
High School:	10 (32%)	13 (33%)	
Some College:	18 (58%)	16 (41%)	
College & Above:			

* Data presented as N (%) or Mean (SD) or Median (25th quartile, 75th quartile)

** From t-test for means, Wilcoxon two-sample test for medians, and chi-square test for proportions

**Table 2:** LSmean (SE) CES-DC, STAIC, CBCL scores stratified by GDM Exposure

Mental Health Outcome	Unexposed Mean (SE)	GDM-exposed Mean (SE)	p-value
**Model 1: Adjusted for child age, sex, socioeconomic status and maternal pre-pregnancy BMI.**			
**Depressive symptoms**	2.29 (0.19)	2.75 (0.16)	0.07
**Anxiety symptoms**	4.23 (0.20)	4.79 (0.16)	**0.03**
**Internalizing symptoms**	1.45 (0.20)	1.99 (0.16)	**0.04**
**Externalizing symptoms**	1.43 (0.21)	1.33 (0.17)	0.69
**Model 2: Further adjusted for maternal race and ethnicity**			
**Depressive symptoms**	2.39 (0.20)	2.69 (0.16)	0.23
**Anxiety symptoms**	4.29 (0.21)	4.75 (0.17)	0.09
**Internalizing symptoms**	1.52 (0.21)	1.91 (0.17)	0.11
**Externalizing symptoms**	1.45 (0.22)	1.32 (0.17)	0.63

Abbreviations: Center for Epidemiological Studies Depression Scale for Children, CES-DC. State-Trait Anxiety Inventory for Children, STAIC. Child Behavior Checklist (CBCL). Least-Square Mean (LSMean) transformed by respective standard deviations.

**Table 3:** LSmean (SE) CES-DC, STAIC, CBCL scores stratified by MVPA levels and GDM Exposure

Mental Health Outcome	Unexposed Mean (SE)	GDM-exposed Mean (SE)	p-value
	Low MVPA Group		
**Depressive symptoms**	2.46 (0.23)	3.16 (0.22)	**0.04**
**Anxiety symptoms**	4.26 (0.20)	5.15 (0.19)	**0.004**
**Internalizing symptoms**	1.67 (0.30)	2.56 (0.28)	**0.04**
**Externalizing symptoms**	1.13 (0.28)	1.67 (0.27)	0.19
	High MVPA Group		
**Depressive symptoms**	2.34 (0.33)	2.39 (0.22)	0.90
**Anxiety symptoms**	4.42 (0.37)	4.55 (0.25)	0.78
**Internalizing symptoms**	1.50 (0.29)	1.44 (0.19)	0.86
**Externalizing symptoms**	1.99 (0.34)	1.04 (0.23)	**0.03**

Transformed LSmean Depressive Symptoms A), Anxiety Symptoms B), Internalizing symptoms C) and Externalizing Symptoms D) adjusted for child age, sex, socioeconomic status, maternal pre-pregnancy BMI and maternal race and ethnicity. Moderate to vigorous physical activity (MVPA). Low MVPA group engaged in less than the recommended amount of MVPA for children, high MVPA group engaged in more than recommended amount of MVPA for children. Low MVPA unexposed (N = 13), Low MVPA GDM-exposed (N = 15), high MVPA unexposed (N = 14), high MVPA GDM-exposed (N = 19).

## Data Availability

Data available upon request.

## Abbreviations

GDM: gestational diabetes mellitus
MVPA: moderate-to-vigorous physical activity
CES-DC: Epidemiological Studies Depression Scale for Children
STAIC: State-Trait Anxiety Inventory for Children
CBCL: Child Behavior Checklist
3DPAR: 3-day physical activity
BDNF: derived neurotropic factor
IGF-1: insulin-like growth factor one
EMR: electronic medical records
KPSC: Kaiser Permanente Southern California
USC: University of Southern California
